# ALX4, an epigenetically down regulated tumor suppressor, inhibits breast cancer progression by interfering Wnt/β-catenin pathway

**DOI:** 10.1186/s13046-017-0643-9

**Published:** 2017-11-28

**Authors:** Juntang Yang, Fei Han, Wenbin Liu, Hongqiang Chen, Xianglin Hao, Xiao Jiang, Li Yin, Yongsheng Huang, Jia Cao, Huidong Zhang, Jinyi Liu

**Affiliations:** Institute of Toxicology, College of Preventive Medicine, Third Military Medical University, 30 Gaotanyan Street, Shapingba District, Chongqing, 400038 People’s Republic of China

**Keywords:** ALX4, DNA methylation, Wnt/β-catenin and breast cancer

## Abstract

**Background:**

ALX4 is a paired-like homedomain transcription factor mainly expressed in the mesenchymal compartment of variety of developing tissues, but its functions, regulation mechanisms and clinical values in breast cancer remains unclear.

**Methods:**

The expression of ALX4 in breast cancer cell lines and patients’ tissues were detected by RT-PCR, qPCR and western blot. Furthermore TCGA database was applied to confirm these results. MSP and BSP methods were used to assess the methylation of ALX4 promoter region. In vitro proliferation, metastasis and in vivo nude mice model were used to evaluate the anti-tumor effect of ALX4 on breast cancer cell lines. Luciferase reporter assay, western blot and TCGA database were used to investigate the tumor suppression mechanisms of ALX4. TMA of 142 breast patients was generated to evaluate the clinical significance of ALX4.

**Results:**

Expression analysis revealed that ALX4 expression is down regulated in breast cancer cell lines and tissues. MSP study showed that the promoter region of ALX4 was hyper-methylated 100% (3/3) in breast cancer cell lines and 69.44% (75/108) in primary breast tumors tissues while 0% (0/8) in normal breast tissues. 5-aza-dc de-methylation treatment restored ALX4 expression in breast cancer cell lines. Functional studies showed that ectopic expression of ALX4 in breast cancer cells inhibited cell proliferation, metastasis in vitro and in vivo. Mechanism study found that ALX4 exerted its anti-tumor function by suppressing the Wnt/β-catenin pathway through promoting the phosphorylation degradation of β-catenin in a GSK3β dependent manner. Clinically multivariate analysis showed that ALX4 expression was an independent favorable prognostic factor in breast cancer patients.

**Conclusions:**

We reveal for the first time that ALX4 acts as a novel functional tumor suppressor inactivated by DNA methylation and is an independent prognostic factor in breast cancer.

**Electronic supplementary material:**

The online version of this article (10.1186/s13046-017-0643-9) contains supplementary material, which is available to authorized users.

## Background

According to the latest survey, breast cancer is the most frequently diagnosed and the leading cause of cancer related death among female worldwide [1, 2]. Despite the dramatic progress achieved in diagnostic and treatment techniques, the prognosis for breast cancer patients has not increased significantly [3, 4]. To improve the survival rate of breast cancer patients, novel strategies especially molecularly targeted therapies are need to be urgently pursued, thus better understanding of the key molecular changes in normal cells that lead to malignant tumor cells is of great importance. From the molecular perspective, the tumorigenesis of breast cancer is a multi-step process comprised of sequential genetic and epigenetic changes [5, 6]. Despite the loss of heterozygosity and acquire of mutations, accumulating studies indicate that DNA hyper-methylation is the third most common mechanism of the inactivation of tumor suppressor gene [7, 8].

Recently a series of methylation-silenced tumor suppression genes have been reported to be associated with cancer initiation and progression [9, 10]. Thus, the identification of novel functional genes associated with CpG island methylation may help to provide insights into the mechanisms for the inactivation of the tumor suppressive pathways involved in breast cancer and identify better potential targets for the diagnosis and treatment.

ALX4 is a paired-like homedomain transcription factor [11] mainly expressed in the mesenchymal compartment of variety of developing tissues [11–17]. We have previously reported that ALX4 could suppress lung cancer progression by inducing cell apoptosis [18]. Recent studies showed its tumor-promoting or tumor-suppressive roles in HCC and ovarian cancers, indicating its diverse functions among different types of cancer [19, 20]. Previous mice model study demonstrated that ALX4 is required for normal mammary gland morphogenesis during mouse puberty and lost expression in stromal and epithelial cells in breast tumors [13, 14], suggesting it may be involved in the precancerous lesions of breast cancer. In the present study we detected the expression and promoter methylation status of ALX4 in breast cancer cell lines, normal human breast tissues and primary human breast tumor tissues. Furthermore the biological function, molecular mechanisms and clinical significance of ALX4 were investigated in breast cancer.

## Materials and methods

### Cell lines and patient sample

The human breast cancer cell lines MCF-7, MB-MDA-231 and T-47D were obtained from the Cell Bank of the Chinese Academy of Science (Shanghai, China) respectively. All cells were cultured in the corresponding medium (recommended by the suppliers) supplemented with 10% fetal bovine serum, and maintained at 37 °C incubator with 5% CO_2_. A total of 108 primary paraffin embedded breast cancer tissue and 8 normal breast tissues of patients who had mammary anaplasty were collected from the Southwest Hospital affiliated to the Third Military Medical University. All experiments and procedures were approved by the Clinical Research Ethics Committee of the Third Military Medical University.

### Reverse-transcription polymerase chain reaction (RT-PCR) and real-time qRT-PCR analysis

Total RNA was extracted from cells and tissues with Trizol (Invitrogen, Carlsbad, CA, USA) according to the manufacturer’s protocol. RT-PCR and real-time quantitative PCR analyses were performed as previously described [9]. Primer sequences are listed in Additional file 1: Table S1.

### Western blot and SiRNA

WB was performed as previously described [9]. After incubation with the secondary antibody, the proteins were detected by chemiluminescence (Millipore Germany). Primary antibodies used in this study were anti-ALX4 (1:1000; Santa Cruz Biotechnology), anti-CTNNB1 (1:700; Santa Cruz Biotechnology), anti-p-CTNNB1 (1800; Santa Cruz Biotechnology), anti-GSK3β (1:800; Santa Cruz Biotechnology), anti-c-Myc (1:1000; Santa Cruz Biotechnology), anti-CCND1 (1:1000; Santa Cruz Biotechnology), anti-MMP7 (1:1000; Abcam) and anti-GAPDH (1:2000; Beyotime China). The siRNA targeting human GSK3β was purchased from Riobio (stQ0004712–1).

### Methylation analysis of ALX4 CpG islands by methylation-specific PCR (MSP) and bisulfite genomic sequencing (BGS)

DNA samples were modified using the EZ DNA Methylation-Gold Kit (Zymo Research, Orange, CA, USA). The MSP and BGS were performed as previously reported [9, 18]. Primers are listed in Additional file 1: Table S1.

### De-methylation experiments

Cells were treated with 5-aza-2-deoxycytidine (Sigma) as previously described [9].

### MTS and EdU assay

Briefly, 5000 cells per well were seeded in 96-well plates and transfected with pIRES2-EGFP-ALX4, ALX4-miRNA and control vectors respectively. The absorbance was determined one day after cells were plated to confirm the identical cell number of each group. Cell viability was evaluated by with Cell Proliferation Reagent MTS (Promega) according to the manufacturer’s instructions.

### Flow cytometry assay

ALX4 or vector control-transfected cells (3 × 10^5^ cells per well) were harvested at 48-h post-transfection, and fixed in 70% ethanol overnight at 4 °C. The cells were stained with propidium iodide (BD Pharmingen, San Jose, CA, USA). A total of 30,000 cells were sorted by Accuri-C6 (BD Biosciences, Franklin Lakes, NJ, USA) and cell cycle profiles were analyzed using the Flowjo software.

### Tissue microarray (TMA) generation

A total of 142 primary Breast cancer patients who had undergone surgical resection with curative intent between 2004 and 2009, were obtained from the Southwest Hospital in Chongqing, China. The clinico-pathologic information was retrieved from the patients’ electronic medical records including age, gender, tumor size, histological grade, lymph node (negative or positive) and clinical stage (defined according to American Joint Committee on Cancer 7th edition) and follow-up information (5–10 years) for overall survival rates. This study was approved by the ethics committee of the Southwest Hospital Affiliated to Third Military Medical University, and all experiments were carried out in accordance with approved guidelines of Third Military Medical University. Informed consent was signed by all of the recruited patients. All samples from breast cancer patients were reviewed histologically by hematoxylin and eosin staining. To construct the TMA slides, two cores were taken from each representative tumor tissues (within a distance of 20 mm). The tissues were stained with hematoxylin-eosin and then reviewed histologically by at least two pathologists. Duplicate cylinders from intratumoral and peritumoral areas were obtained. Finally, the TMAs were constructed (in collaboration with Shanghai Biochip Company Ltd., Shanghai, China).

### Immuno-histochemical (IHC) analysis and scoring

IHC staining was performed using the antibody against ALX4 as described previously [21]. The staining was evaluated for the tumor cells. The immunostaining was considered positive when ≥10% of the tumor cells being immunoreactive. A double-blind method was carried out independently by two investigators without knowing the patients’ clinical and pathological features to analyze the IHC results. Three visual fields from different areas of each specimen were chosen randomly for the IHC evaluation. ALX4 expression was scored according to staining intensity and the percentage of positive cells as previously described [22]. The percentage of positive cells was scored as follows: 0% -10% (0), 11%–30% (1), 31%–50% (2), 51%–80% (3) and 81%–100% (4). Staining intensity was scored as follows: no staining (0), week (1), moderate (2), and strong (3). Comprehensive score = staining percentage × intensity. ALX4 expression was classified as follows: ≤4 low expression, >4 high expression according to the median of 142 breast cancer patients.

### Generation of stable cell lines

The ALX4 over expression or knock down cell lines were generated as previously described [9, 21]. Briefly the PIRES2-EGFP-ALX4 vector or the pcDNA6.2-GW/EmGFP-miR vector was transfected using ViaFect transfection reagent. The stably transfected cells were screened with G418 (Calbiochem, La Jolla, CA, USA) or Blasticidin (Invitrogen Preservation). Single clone was obtained by the cylinder method. Several positive clones were confirmed by qPCR and then mixed for consequent experiments.

### Colony formation assay

MDA-MB-231 and MCF-7 cells were plated in 6-well plates at 2.5 × 10^5^ cells per well. For knockdown, MDA-MB-231-ALX4 and MCF-7-ALX4 stably cells were plated in 6-well plates at 3 × 10^5^ cells per well. After culturing for 24 h, cells were transfected with ALX4 or vector control and miRNA or negative control, respectively. After 48 h of transfection, cells were collected, diluted 1:3, plated in 6-well plates and selected with 0.8 mg/ml of G418 or 2 μg/ml of Blasticidin for 14 days to establish stable clones in which the plasmids had stably integrated into genomic DNA. Surviving colonies (⩾50 cells per colony) were stained by using crystal violet (Sigma-Aldrich, St Louis, MO, USA) and counted.

### Cell migration and invasion assay

For migration assay, cells (4 × 10^4^/ well) were suspended in 300 μL serum-free medium and seeded in the upper transwell chamber (8 μm pore size, BD Biosciences). For invasion assay, cells in serum-free medium were placed into the upper chamber of an insert coated with Matrigel (BD Biosciences). After incubation for 12 h at 37 °C, non-migrated or non-invaded cells on the upper membrane were removed by a cotton swab. Cells that had migrated or invaded through the membrane were stained with 0.1% crystal violet and three fields were randomly selected for cell number counting.

### Animal experiments

BALB/c-nude mice (4–5 weeks of age, 18–20 g) were purchased from the Center of Experimental Animal of Third Military Medical University, China and housed in a sterile environment. For tumor formation experiment six 4-week-old female BALB/c-nude mice were randomly divided into two groups (*n* = 3/group). MDA-MB-231 cells (8× 10^6^) with ALX4 or control vector stably expression were injected subcutaneously into the right flanks of the nude mice, respectively. The tumor volume was determined using the eq. V = 0.5 × D × d^2^ (V, volume; D, longitudinal diameter; d, latitudinal diameter). The developing tumors were observed over the next 33 days. For metastasis experiment six 4-week-old female BALB/c-nude mice were randomly divided into two groups (n = 3/group) and MDA-MB-231 cells (6× 10^6^) with ALX4 or control vector stably expression were injected via tail veins, respectively. Tumor metastasis were observed by H&E staining and qualified by human-specific β_2_-MG (beta-2-microglobulin) [23] 5 weeks later. All experimental animal procedures were approved by the Institutional Animal Care and Use Committee of Third Military Medical University, China.

### TOP/FOP flash reporter assays

The previously reported promoter region for CTNNB1 (−2760/+27) was amplified and cloned into the pGL3 vector (Promega, Madison, WI, USA) [24]. MDA-MB-231 and MCF-7 cells were transfected with the pGL3-CTNNB1/promoter together with pIRES2-EGFP-ALX4 or control vector and pRTK-Luc (Renilla-TK-luciferase vector, Promega) to normalize the transfection efficiency. 36 h later, the activities of Firefly luciferase and Renilla luciferase were measured using the Dual Luciferase Reporter Assay System (Promega). For TOP and FOP flash assay, MDA-MB-231 and MCF-7 cells were plated into 24-well plates at a concentration of 2.0 × 10^4^ cells per well. Cells were co-transfected with 300 ng of either TOP flash (T-cell factor reporter plasmid) or FOP flash (mutant T-cell factor reporter plasmid) expression plasmids (Millipore, Temecula, CA, USA), and 300 ng of pIRES2-EGFP-ALX4 or control vector and 30 ng pRL-TK. Luciferase activity was measured in triplicate using the fluorescence microplate reader measurement system Varioskan LUX (Thermo Fisher, Waltham, MA, USA) using a Dual-luciferase reporter kit (Promega) as previously described [25].

### Analysis of publicly available datasets

TCGA dataset (https://genome-cancer.soe.ucsc.edu/proj/site/xena) was applied to analysis the relationship between ALX4 expression prognostic outcomes of breast cancer patients. Log in the website and click “launch Xena Browser” and in the first section “Select a Study to Explore”, select “TCGA breast cancer (BRCA)” data set in the case of our study. In the second section “Select Your First Variable”, select “Phenotypic” for the data type and “sample type” for the Phenotype. In the third section “Select Your Second Variable”, select “Genomic” for the data type and input the gene name “ALX4” then select Gene expression. When all the parameters are selected we will proceed to the next page and input “Primary tumor” in the filter actions and click “Filter”. Finally click “Kaplan Meier plotter” the relationship between ALX4 expression prognostic outcomes of breast cancer patients will be showed.

The TCGA breast cancer RNAseq (IlluminaHiSeq; *N* = 1124) data was applied to analyze the expression of ALX4 and genes related to Wnt/β-catenin signaling. The heat maps of gene expression were generated using https://genome-cancer.ucsc.edu/proj/site/hgHeatmap/. First click “add datasets” on the left corner and the select “TCGA breast cancer RNAseq (IlluminaHiSeq; N = 1124)”. By inputting the gene names the expression heap map will be generated automatically. For statistically analysis, down load the raw data in “.tgz” form and open it with Excel software and analyze the data with relative statistical methods.

### Statistical analysis

Statistical analyses were performed using the SPSS 16.0 software (SPSS, Inc., Chicago, IL). Data were expressed as means ± standard deviation (SD). Kaplan-Meier survival plots and the Cox regression methods were used to compare the survival outcome between two ALX4 expression groups. The relationship between ALX4 expression and clinical-pathological parameters was analyzed by Chi-square test and Linear-by-Linear Association (2-sided). *P*-value <0.05 was considered to denote statistical significance.

## Results

### ALX4 expression is down regulated in breast cancer cell lines and tissues

**Fig. 1:**
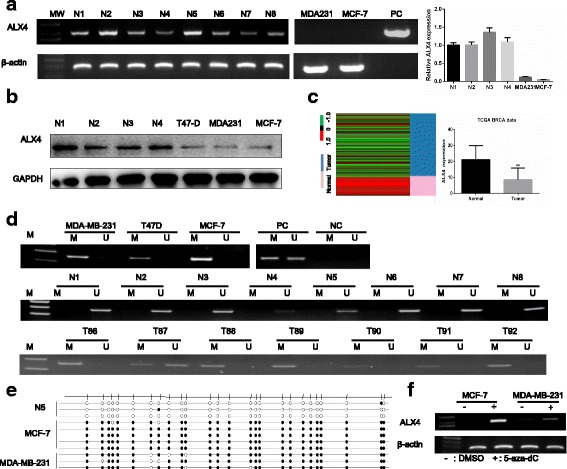
The down regulation of ALX4 in breast cancer is caused by promoter methylation. **a** Analysis of ALX4 mRNA expression levels in normal breast tissues and breast cancer cells using RT-PCR and qPCR. ALX4 was down regulated in breast cancer cells compared with normal breast tissues. **b** The protein expression of ALX4 was detected in normal breast tissues and breast cancer cells. **c** Down regulation of ALX4 in breast cancer patients was further confirmed using TCGA database. ALX4 mRNA expression in the TCGA breast cancer RNAseq (IlluminaHiSeq; *n* = 1208, normal tissue = 111, breast cancer tissue = 1097) data sets. Error bars indicate s.d. ** *P* < 0.01. **d** ALX4 promoter methylation was analyzed by MSP method. ALX4 gene promoter was methylated in breast cancer cell lines, unmethylated in all normal breast tissues and frequently methylated in breast tumor tissues. M: PCR product amplifed by methylated -specific primers; U: PCR product amplifed by unmethylated specific primers; N: normal tissue; T: tumor tissues; PC: positive control, including fully unmethylated or fully methylated control; NC: negative control. **e** Methylation status of ALX4 promoter region of normal breast tissue and breast cancer cell lines was analyzed by BSP method. CpG islands were represented as open dots (unmethylated) or filled dots (methylated). **f** Re-expression of ALX4 in MDA-MB-231 and MCF-7 cell lines after treatment by pharmacologic demethylation. −: DMSO control; +: 5-aza-dC

The expression status of ALX4 in breast cancer cell lines and normal breast tissues were detected by RT-PCR, qRT-PCR and WB. The results showed that ALX4 was down regulated in breast cancer cell lines compared with the normal breast tissues both on mRNA and protein level (Fig. 1a, b). To further confirm this observation, the expression pattern of ALX4 was subsequently analyzed using The Cancer Genome Atlas (TCGA) database (111 normal breast tissue and 1097 breast cancer tissue). Consistent with our results, ALX4 expression was down regulated in TCGA breast cancer tissues compared with normal breast tissues (*P* < 0.01) (Fig. 1c). Collectively these results suggested that ALX4 was down regulated in breast cancer, thus it may play an important role in breast cancer development.

### Methylation of ALX4 is associated with its down regulation in breast cancer

Our previous study on lung cancer has identified numbers of CpG islands on the ALX4 promoter region [18] and epigenetically silencing by promoter hyper-methylation have been reported to be a major reason for gene down regulation [25–27]. To investigate whether the expression of ALX4 was associated with promoter methylation in breast cancer, the methylation status of the ALX4 promoter region was firstly detected by MSP. Complete methylated status was observed in three breast cancer cell lines MDA-MB-231, MCF-7 and T47D (Fig. 1d). We further examined the methylation of clinical samples and the results showed that the methylation rate of the promoter region of ALX4 gene was 69.44% (75/108) in breast cancer tissues of patients, and 0% (0/8) in 8 normal breast tissues (Fig. 1d). To further validate the above results, we used the BSP method to quantitatively detect the methylation status of ALX4 promoter in normal breast tissue and two commonly used breast cancer cell lines. The results showed that CpG island methylation was not detected in the randomly selected normal breast tissue, while significant CpG island methylation was detected in the two breast cancer cell lines MDA-MB-231 and MCF-7 (Fig. 1e). These above results demonstrated that the promoter region of ALX4 exhibited hyper-methylation status in breast cancer. To investigate the relationship between low expression and methylation status of ALX4, we first analyzed the TCGA data using cbioportal website (www.cbioportal.com). After downloading the original data, we analyzed the relationship between methylation and expression, and found the expression of ALX4 gene was negatively correlated with the methylation degree (*P* < 0.00) (Additional file: Fig. S1). In order to further verify that down regulation of ALX4 in breast cancer is related to methylation, breast cancer cell lines were treated with 5-aza-dc, a pharmacological inhibitor of DNA methylation as previously described [9, 28] and the results showed that ALX4 expression was restored (Fig. 1f). These results indicated that promoter methylation is responsible for the down regulation of ALX4 in breast cancer.

### ALX4 over expression inhibits breast cancer cell proliferation, migration and invasion in vitro

**Fig. 2:**
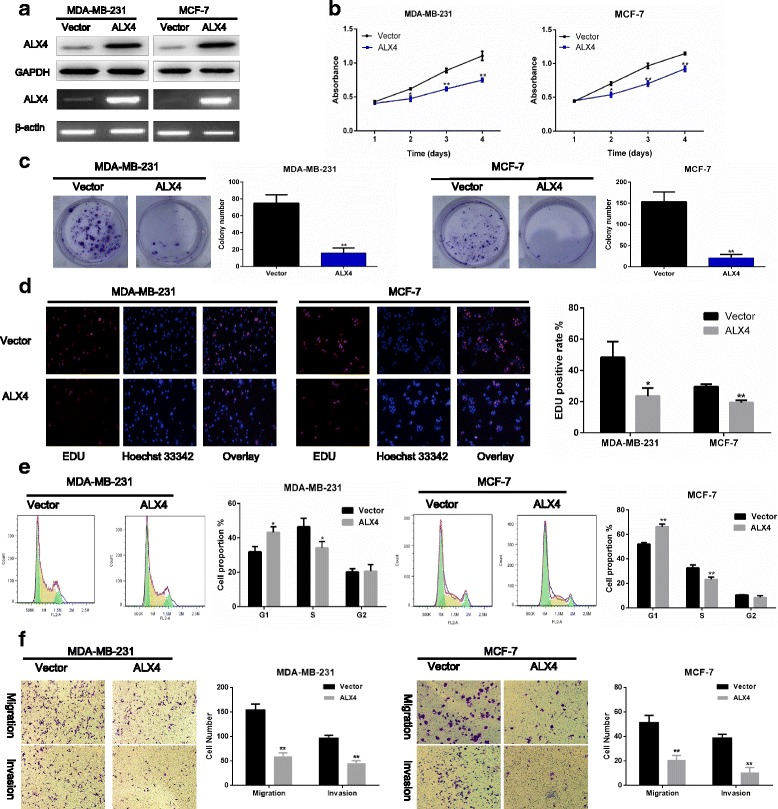
ALX4 suppresses breast cancer cell proliferation and metastasis in vitro. **a** Transfectants of the control vector and ALX4 were identified WB and RT-PCR in MDA-MB-231 and MCF-7 cell lines. **b** MTS assays were performed to examine the effect of ALX4 over expression on the relative number of viable cells based on the absorbance. **c** Over expression of ALX4 reduced the colony formation ability of MDA-MB-231 and MCF-7 cell lines. Error bars indicate s.d. (*n* = 3).* *P* < 0.05, ** P < 0.01. **d** EDU assay was performed to detect the effect of ALX4 over expression on proliferation. **e** Cell cycles were analyzed after ALX4 over expression using PI stainning. **f** Transwell assays were used to examine the effect of ALX4 on cell migration and invasion in MDA-MB-231 and MCF-7 cell lines

To investigate the function of ALX4 in breast cancer, we first transfected MDA-MB-231 and MCF-7 cell lines using control vector and ALX4 expression vector and verified the expression of ALX4 by WB and RT-PCR (Fig. 2a). The effects of ALX4 over expression on cell proliferation and viability were firstly detected. A four days growth curve showed that ALX4 over expression inhibited the proliferation of MDA-MB-231 and MCF-7 cells (Fig. 2b). The suppressive function of ALX4 was further verified by colony formation assay and EDU assay. ALX4 over expression inhibited the colony formation and the EDU positive rate of MDA- MB-231 and MCF-7 cell lines (Fig. 2c, d). Furthermore, we found that ALX4 over expression induced G1 / S blockade of breast cancer cell lines (Fig. 2e). We further investigated the effect of ALX4 on breast cancer cell metastasis. Transwell assays with or without matrix gel showed that ALX4 suppressed the migration and invasion ability of MDA-MB-231 and MCF-7 (Fig. 2f). These data suggested that ALX4 inhibited the proliferation and metastasis of breast cancer cell lines.

### Knockdown of ALX4 expression recover cell proliferation, migration and invasion of breast cancer cells

**Fig. 3:**
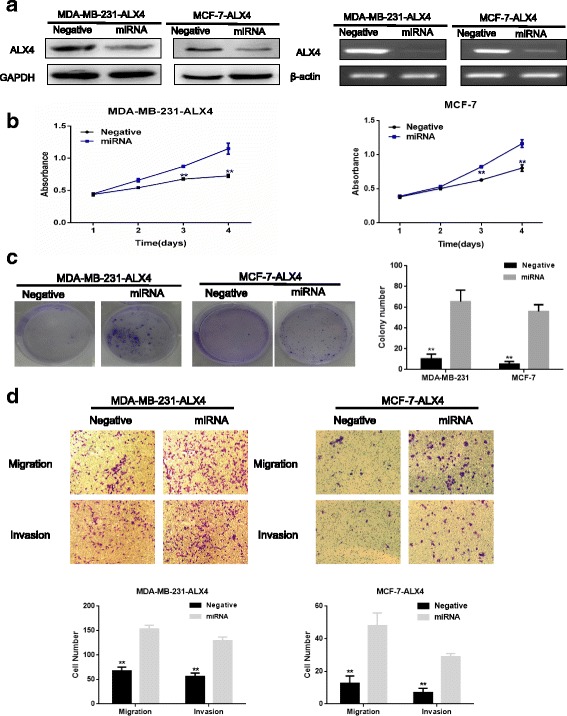
Knockdown of ALX4 in MDA-MB-231-ALX4 and MCF-7-ALX4 stably cells recovered the inhibition effect. **a** Knockdown of ALX4 in MDA-MB-231-ALX4 and MCF-7-ALX4 stably cells was identified by WB and RT-PCR. **b** MTS assays were used to examine the effect of ALX4 knockdown on the relative number of viable cells based on the absorbance. **c** Knockdown of ALX4 recovered the colony formation ability. **d** Transwell assays were performed to examine the effect of ALX4 knockdown on cell migration and invasion. Error bars indicate s.d. (n = 3).* *P* < 0.05; ** *P* < 0.01

To further confirm the role of ALX4 in breast cancer cell growth and metastasis. We knocked down ALX4 expression in the MDA-MB-231-ALX4 and MCF-7-ALX4 stably cell lines with ALX4-miRNA. ALX4 expression was reduced in cells transfected with ALX4-miRNA as shown by WB and RT-PCR (Fig. 3a). Knockdown of ALX4 markedly enhanced cell viability and colony formation ability compared with the control group (Fig. 3b, c). Furthermore, knockdown of ALX4 reversed the inhibitory effect on cell metastasis of MDA-MB-231-ALX4 and MCF7-ALX4 stably cells (Fig. 3d). These knockdown results, together with the above obtained data from ALX4 over expression study suggested that ALX4 might function as a potential tumor suppressor in breast cancer.

### Overexpression of ALX4 inhibits tumor formation and metastasis in nude mice

**Fig. 4:**
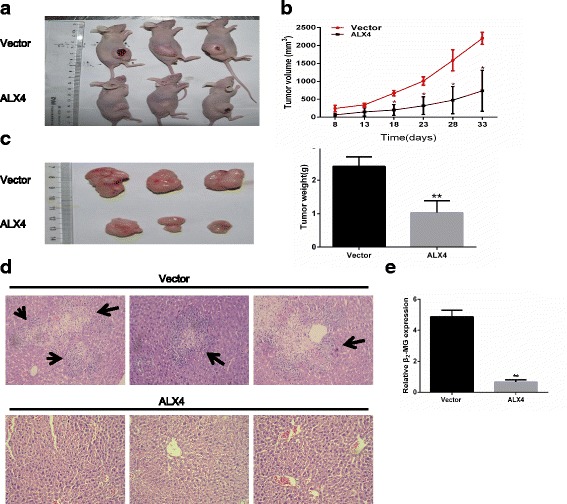
ALX4 inhibits tumor formation and metastasis in nude mice. **a** Control vector and ALX4 stably expressing MDA-MB-231 cells (8 × 10^6^) were subcutaneously injected into the right flank of nude mice. Pictures of BALB/c-nude mice and solid tumor tissues were taken after 33 days. **b** The tumor growth curve of ALX4 over expressing cells was compared with vector control cells. **c** Tumor weights in the vector control and ALX4 groups were determined. Error bars indicate s.d. (n = 3). **d** Liver metastases were observed by H&E staining in the control group but not in the ALX4 group by tail veins injection method. Arrows indicate the metastatic loci. **e** Liver metastasis was further quantified using RT-qPCR. Human-specific β_2_-MG levels were used to quantify metastatic human cancer cells with the mouse-specific β_2_-MG level as an internal control. The nude mice that were injected with ALX4 over expressing cells demonstrated a significantly lower number of metastatic cancer cells in the liver compared with those that were injected with an empty control. Error bars indicate s.d. (n = 3).*P < 0.05; **P < 0.01

To further investigate the tumor suppression function in vivo, nude mice xenograft tumor model was applied to determine the oncogenic role of ALX4 in tumorigenicity of breast cancer cells. MDA-MB-231-ALX4 and vector control stably over expression cells were subcutaneously injected into nude mice to evaluate the effect on tumor formation. The result showed that mice receiving MDA-MB-231-ALX4 stably cells exhibited significantly reduced tumor formation ability compared with vector control cells (Fig. 4A-C). Furthermore, we investigated the effect of ALX4 over expression on breast cancer cells metastasis in vivo via tail veins injection method. H&E staining results showed that ALX4 could suppress the liver metastasis (Fig. 4D) and this observation was further confirmed by qPCR using human-specific β_2_-MG (beta-2-microglobulin) [23] with mouse-specific β_2_-MG as internal control (Fig. 4E). Collectively, these data indicated that ALX4 had a significant effect on impeding tumor formation and metastasis, supporting ALX4 as a tumor suppressor in breast cancer in vivo.

### ALX4 exerts its anti-cancer function via Wnt/β-catenin pathway by degradation of β-catenin in breast cancer

**Fig. 5:**
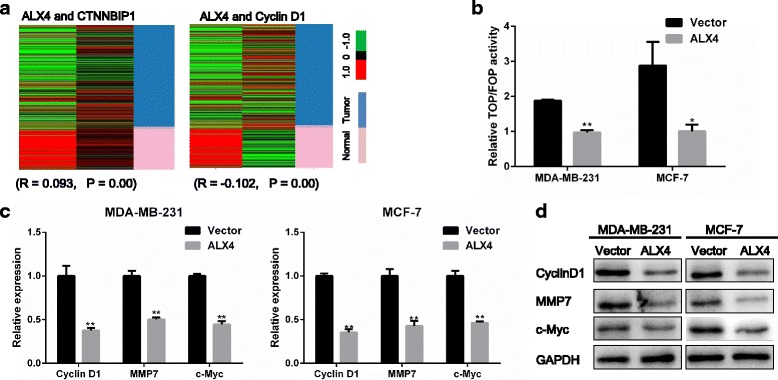
ALX4 suppresses the Wnt/β-catenin pathway. **a** ALX4 was positively correlated with CTNNBIP1 expression (*R* = 0.093, *P* = 0.00) and negatively correlated with Cyclin D1 expression (*R* = −0.102, P = 0.00). **b** ALX4 decreased the transcriptional activity of the Wnt/β-catenin pathway as determined by a TOP flash/FOP flash reporter luciferase activity assay in MDA-MB-231 and MCF-7 cell lines. TOP and FOP flash plasmids were co-transfected with control vector or ALX4 and PRL-TK plasmids in MDA-MB-231 and MCF-7 cells. **c, d** ALX4 inhibited the expression of Wnt/β-catenin pathway target genes at both the mRNA and protein levels. Error bars indicate s.d. (n = 3). *P < 0.05; **P < 0.01

**Fig. 6:**
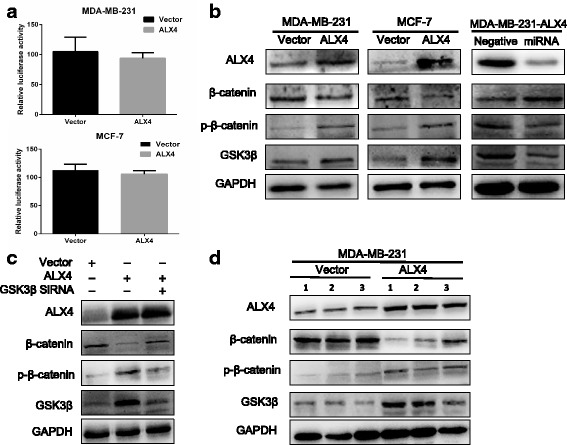
ALX4 represses the Wnt/β-catenin pathway by promoting the phosphorylation degradation of β-catenin. **a** The full length human β-catenin promoter (−2760 bp to +27 bp) was cloned into the PGL3-Basic luciferase reporter vector. The β-catenin transcription activity was examined after ectopic expression of ALX4 in MDA-MB-231 and MCF-7 cell lines. **b** ALX4 promoted the phosphorylation degradation of β-catenin in breast cancer cell lines. **c** Knock down of GSK3β by siRNA in MDA-MB-231-ALX4 cells decreased the p-β-catenin and recovered the protein level of β-catenin. **d** The protein expression of β-catenin, p-β-catenin and GSK3β were detected in xenograft tumors

**Fig. 7:**
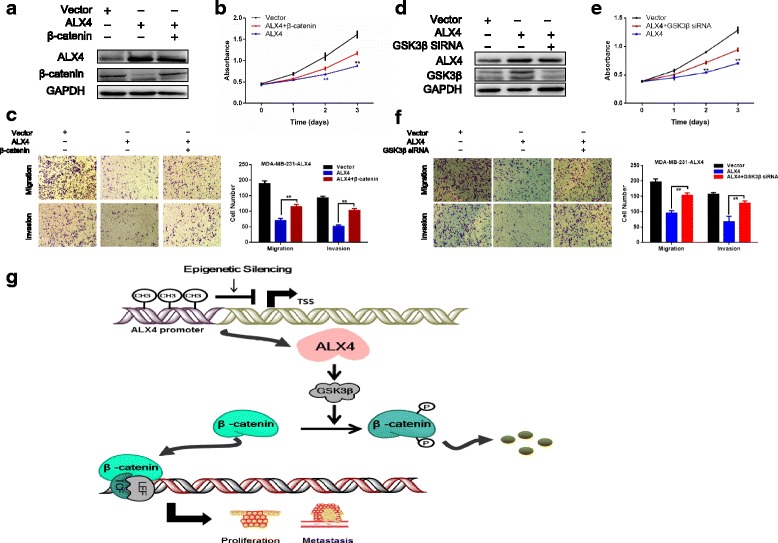
ALX4 inhibited the progression of breast cancer via promoting phosphorylation degradation of β-catenin in a GSK3β dependent manner. **a** The expression of ALX4 and β-catenin was analyzed by WB 48 h after transfection with control vector or β-catenin over expression vector in MDA-MB-231-ALX4 cells. **b** MTS assays were used to examine the effect of β-catenin re-expression on cell proliferation in MDA-MB-231-ALX4 cells. **c** Transwell assays were used to examine the effect of β-catenin re-expression on cell metastasis of MDA-MB-231-ALX4 cell. **d** The expression of ALX4 and GSK3β was analyzed by WB 48 h after transfection with control vector or GSK3β siRNA vector in MDA-MB-231-ALX4 cells. **e** MTS assays were used to examine the effect of GSK3β knock down on cell proliferation in MDA-MB-231-ALX4 cells. **f** Transwell assays were used to examine the effect of GSK3β knock down on cell metastasis in MDA-MB-231-ALX4 cell. **g** Schematic diagram of the mechanisms of ALX4 mediated suppression of breast cancer cell proliferation and metastasis based on our study. ALX4 reduces the protein level of β-catenin by promoting its phosphorylation degradation via up regulation of GSK3β, which subsequently inhibits the activation of Wnt/β-catenin signaling

We next went to explore the possible mechanism by which ALX4 suppressed breast cancer progression. Accumulating evidence suggested that abnormally activated Wnt/β-catenin pathway contributed to the malignant phenotype of breast cancer [29–31]. Using the TCGA data base, we found that ALX4 expression was positively correlated with the negative regulator of Wnt/β-catenin pathway, such as WIF1 and CTNNBIP1, but negatively correlated with Wnt/β-catenin activated genes, LEF1 and Cyclin D1 (Fig. 5a) (Additional file 1: Figure S2 A, B). In light of this observation, we hypothesized that ALX4 may exerted its inhibitory function by disrupting the Wnt/β-catenin signal. To test this presumption, we first performed Top/Fop flash reporter assay, the results showed that ALX4 could significantly inhibit the transcription activity of β-catenin/T-cell factor (TCF) compared with the vector control group in MDA-MB-231 and MCF-7 cell lines (Fig. 5b). We subsequently analyzed the expression of the downstream target genes of Wnt/β-catenin pathway, and found that ALX4 could decrease the mRNA and protein level of CyclinD1, c-Myc and MMP7 (Fig. 5c, d). Next we thought to figure out the possible mechanism by which ALX4 could interrupt the Wnt/β-catenin signaling. As a transcription factor, we first examine the effect of ALX4 over expression on the transcription of β-catenin (the key signal transmitter involved in Wnt/β-catenin pathway). However, luciferase reporter assay showed that ALX4 had no significant inhibition effects on the transcription of β-catenin in the two breast cancer cell lines (Fig. 6a). Furthermore no significant change was observed for the mRNA expression of β-catenin after ALX4 overexpression (data not shown). Nevertheless we found that the protein level of β-catenin was decreased after ALX4 expression (Fig. 6b). Accumulating evidence indicated that the protein level of β-catenin was tightly controlled by the degradation complex composed by AXIN, ICAT, APC and GSK-3β by which GSK-3β phosphorylates β-catenin leading to its proteolytic degradation [32–35]. Thus we speculated that ALX4 may promote the phosphorylation degradation of β-catenin. To test this hypothesis, we detected the relative protein expression and the results showed that ALX4 induced the level of p-β-catenin and GSK-3β (Fig. 6b). To further investigate whether GSK3β play important role in ALX4 mediated β-catenin phosphorylation and degradation, we knocked down GSK3β expression in MDA-MB-231-ALX4 stably cell line using siRNA and the level of p-β-catenin was reduced while the protein level of β-catenin was recovered (Fig. 6c). We further consolidated our observation by detecting the expression of ALX4, β-catenin, p-β-catenin and GSK-3β in xenograft tumor tissues by WB (Fig. 6d). Furthermore, by analyzing the TCGA breast cancer data, we found that the expression of ALX4 was positively correlated with the expression of the key members of the “β-catenin degradation complex” (Additional file 1: Figure S2C). To define whether β-catenin was required for the anti-tumorigenesis function of ALX4, we re-expressed β-catenin in MDA-MB-231-ALX4 stably cell line (Fig. 7a). β-catenin re-expression reversed the inhibitory effect of ALX4 on cell proliferation and metastasis (Fig. 7b, c). These data indicated that β-catenin was important for the tumor suppression function of ALX4 in breast cancer. We further examine the effect of GSK3β knock down on the phenotypes of MDA-MB-231-ALX4 stably cell line. Knock down of GSK3β was confirmed by WB (Fig. 7d), and the results showed that knock down of GSK3β could reverse the inhibitory effects of ALX4 on cell proliferation and metastasis at least in part (Fig. 7e, f). Taken together our results supported that ALX4 inhibited the progression of breast cancer through interfering Wnt/β-catenin signaling via promoting phosphorylation degradation of β-catenin in a GSK3β dependent manner (Fig. 7g).

### The low expression of ALX4 is associated with poor survival outcomes of breast cancer patients

**Fig. 8:**
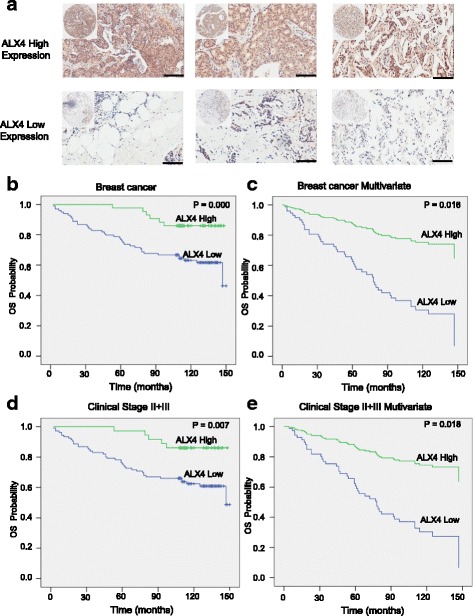
High expression of ALX4 predicts a longer survival time in breast cancer patients. **a** 142 breast cancer patients were divided into two groups according to the expression of ALX4 by identified by IHC. Scale bar, 100 μm. **b** Kaplan-Meier survival analysis of ALX4 expression in breast cancer patients. Long OS was observed in the high ALX4 expression group compared with the low ALX4 group. **c** Multivariate Coxregression analysis of the relationship between ALX4 expression and OS of breast cancer patients. ALX4 was determined to be an independent favorable prognostic factor. **d** Kaplan-Meier survival analysis of ALX4 expression in breast cancer patients at clinical stage II + III. Long OS was observed in the high ALX4 expression group compared with the low ALX4 group. **e** Multivariate Coxregression analysis of the relationship between ALX4 expression and OS of breast cancer patients at clinical stage II + III. ALX4 was determined to be an independent favorable prognostic factor

To investigate the clinical significance of ALX4 expression in breast cancer patients, we first conducted IHC on a TMA containing 142 breast cancer patients with overall survival clinical statistics. After IHC, we used the scoring system to consolidate the results for intensity and positive staining percentage. Based on the results, positive staining of tumor cell was quantified and classified into two groups: high and low according to the median score of 142 breast cancer patients (Fig. 8a). Survival analysis using Kaplan-Meier method and log rank test showed that patients with high ALX4 expression (*n* = 42) presented a longer survival time than that of low ALX4 expression (*n* = 100) (*P* < 0.01) (Fig. 8b). To correct for bias caused by univariate analysis, ALX4 expression as well as other parameters were examined in a multivariate Cox-regression analysis (after adjusting for age, histological grade, clinical stage, tumor size and lymph node status). In addition to tumor size (HR = 1.268, *P* = 0.00), ALX4 expression was found to be an independent prognostic factor (HR = 0.345, *P* = 0.01) for the overall survival of breast cancer patients (Fig. 8c).

To further confirm these results, we performed a meta-analysis of the association between ALX4 gene expression and prognostic outcomes among 1080 breast cancer patients using the TCGA data base. The results showed that high expression of ALX4 predicted longer survival time (*n* = 1080, *p* = 0.00, 2 group; n = 1080, *p* = 0.03, 3 group) (Additional file 1: Figure S4). Next, we sought to determine the impact of ALX4 expression on patient survival considering tumor size, clinical stage, lymph node and histological grade. After stratifying patients based on ALX4 expression, we analyzed patients’ survival data and found a significant association between ALX4 expression and patients at clinical stages II + III (P = 0.00) (Fig. 8d, e). We further investigated the relationship between ALX4 expression and clinical parameters, and found that ALX4 expression was evidently correlated to clinical stages (*n* = 140, P = 0.01), tumor size (*n* = 142, P = 0.01) and lymph node status (*n* = 138, P = 0.00) of breast cancer patients (Table 1). Taken together our data showed that ALX4 is an independent favorable prognostic factor for breast cancer patients and its expression is associated with clinical parameters.

**Table 1:** Correlation between ALX4 expression and clinical parameters from TMA data

		ALX4 expression		
Clinical parameter	Total	High	Low	*P* value
Clinical stages				0.012
I	11	9	2	
II	81	21	60	
III	48	12	36	
Histological grade				0.638
1	35	9	26	
2	106	33	73	
3	1	0	1	
Lymph node status				0.000
Negative	50	25	25	
Positive	88	17	71	
Tumor size				0.016
< 3 cm	48	21	27	
≧3 cm	93	21	72	
Her2				0.264
Positive	43	16	27	
Negative	88	24	64	
PR				0.058
Positive	77	19	58	
Negative	52	21	31	
ER				0.975
Positive	88	27	61	
Negative	42	13	29	

## Discussion

Homeobox genes are a family of genes that share highly conserved structure while maintaining a high degree of diversity [36, 37]. Its conserved sequences encode proteins containing homologous domains that are capable of binding DNA which endow them with the ability to be involved extensively in the process of embryos, tissues and organs development and human diseases [36, 37]. ALX4, a paired-like homedomain transcription factor, is mainly expressed in the mesenchymal compartment of variety of developing tissues such as skull and limbs [11–17]. Recent studies showed its opposing roles in HCC and ovarian cancers via distinct mechanisms [20, 38] indicating the functions and regulation mechanisms of ALX4 in the progression of different tumor remain largely uninvestigated.

In the present study we firstly showed that ALX4 was down regulated in breast cancer. Using MSP and BSP methods we found that the promoter region of ALX4 was frequently methylated in breast cancer and demethylation treatment could recover the expression of ALX4. These results indicated that hyper-methylation contribute to the down regulation of ALX4 in breast cancer. Studies have shown that DNA methylation patterns in tumourigenesis is comprised of genome-wide hypo-methylation and CpG islands hyper-methylation and its main significance may be the molecular basis for tumor suppressor gene inactivation, proto-oncogene activation and genomic instability [7, 8, 39]. Recently, numbers of epigenetically silenced genes were proved to be tumor suppressor in different types of cancer [9, 25, 40, 41]. These evidences indicted that ALX4 may be involved in the tumorigenesis of breast cancer.

Thus gain and loss of function studies were carried out to examine the function of ALX4 in breast cancer. Ectopic expression of ALX4 induced apoptosis (data not shown) and G1/S blocking thus inhibited the proliferation of breast cancer cells in vitro. Distance invasiveness is another malignant phenotype which contributes to the high mortality of breast cancer [5, 6] and we found that ALX4 could attenuate the metastasis ability of breast cancer cell lines in vitro and in vivo. These data suggested that ALX4 function as a tumor suppressor in breast cancer. Previous study found that ALX4 promoted ovarian cancer invasion by forming a complex with HOXB13 [20] but our data showed that ALX4 inhibited breast cancer metastasis. These results suggested that the function of ALX4 varied depending on different cancer types indicating its important roles in tumorigenesis.

We further determined the possible mechanisms of the tumor inhibition effect of ALX4 in breast cancer. Abnormal activating of Wnt/β-catenin signaling has been recognized as an important mechanism of breast cancer initiation and progression [29–31]. We found that ectopic expression of ALX4 could inhibit the canonical Wnt signaling activity in breast cancer by TOP/FOP flash reporter assay and the Wnt target functional genes MMP7, c-Myc and Cyclin D1 were down regulated both at protein and mRNA level. These results demonstrated that ALX4 suppressed breast cancer progression by inhibiting the Wnt/β-catenin pathway. We have previously showed that ALX4 suppressed lung cancer proliferation by activating the caspase cascade [18] and a recent study showed that ALX4 suppressed the EMT of liver cancer by inhibiting the sonic hedgehog (Shh) pathway [38]. These results indicated that the molecular mechanisms of ALX4 as tumor suppressor may be varied among different cancer types.

We further investigated the possible mechanism by which ALX4 inhibiting the Wnt/β-catenin pathway. In the canonical Wnt/β-catenin signaling, β-catenin functions as a key mediator to activate the expression of Wnt target genes thus promoting cell proliferation and metastasis [42–45] and a previous study found that β-catenin is required for the tumorigenic behavior of breast cancer cells [46]. Our data showed that ectopic expression of ALX4 reduced the protein level of β-catenin but had no significant effects on the transcription of β-catenin. Previous studies have suggested that the protein level of β-catenin is tightly controlled by the destruction complex composed by APC AXIN, ICAT and GSK-3β [32, 44, 47, 48] through which β-catenin is finally phosphorylated by GSK-3β at the Ser33 and Ser37 leading to its proteolytic degradation [49–51]. We found that ALX4 could decrease the protein level of β-catenin by promoting its phosphorylation via upregulating the GSK3β. Accumulating evidence indicated that GSK3β mediated β-catenin phosphorylation is the key step to generate the β-TrCP-binding site for the subsequent degradation [35, 52, 53]. A recent study in HCC also showed that GSK-3β suppresses HCC cell dissociation in vitro by inhibiting Wnt/β-catenin signaling pathway [19]. Furthermore we found that the expression of ALX4 was positively correlated with the expression of the key members of the “β-catenin degradation complex”. Collectively, our data suggested for the first time that ALX4 exerted its inhibitory function by suppressing the Wnt/β-catenin pathway through promoting the phosphorylation degradation of β-catenin in a GSK3β dependent manner.

Accumulating studies have indicated that the different expression pattern of gene showed tight correlation with the clinical progression of cancer patients [22, 25]. Despite the dramatic progress achieved in breast cancer related diagnostic and treatment techniques, the prognosis for breast cancer patients has not increased significantly, thus identification of novel biomarker is in urgent need [3, 4]. Therefore, to evaluate the clinical significance of ALX4 in breast cancer patient, we performed a tissue microarray on 142 breast cancer patients with clinical and survival data. We found that ALX4 expression is an independent favorable prognostic factor and is in tight relationship with clinical stages, tumor size and lymph node status in breast cancer patients. These data suggested that the expression of ALX4 may provide information for predicting the survival of breast cancer patients.

## Conclusions

In summary, our study indicates for the first time that ALX4 is an epigenetically inactivated tumor suppressor in breast cancer acting through inhibition the Wnt/β-catenin pathway. This adds to our current knowledge of the tumorigenesis process of breast cancer. Furthermore the expression of ALX4 is an independent favorable prognostic factor in breast cancer patients and is in tightly relationship with tumor progression. This may provide a new biomarker to predict the survival of breast cancer patients.

## Additional file

Additional file 1: Figure S1. The correlation between ALX4 expression and methylation was analyzed using TCGA data set (www.cbioportal.com). A: Cohort1:TCGA, Cell 2015, *n* = 552;Cohort2. B: TCGA, Provisional 2012, *n* = 656 **Figure S2.** Analysis the relationship between ALX4 and Wnt/β-catenin suppression and activation genes in breast cancer using TCGA Database. (A) ALX4 was positively correlated with WIF1 expression (*R* = 0.503, *P* = 0.00). (B) ALX4 was negatively correlated with LEF1 expression (*R* = −0.062, *P* = 0.03). (C) AXL4 was positively correlated with the expression of the key members of “β-catenin degradation complex” **Figure S3.** The illustration of full length human β-catenin promoter (−2760 bp to +27 bp) that was cloned into the PGL3-Basic luciferase reporter vector **Figure S4.** Relationship between ALX4 expression and survival of breast cancer patients retrieved from public datasets(https://genome-cancer.soe.ucsc.edu/proj/site/xena). (A) Survival analysis using TCGA dataset, the results showed that high expression of ALX4 predicted longer survival time (*n* = 1080, *p* = 0.00, 2 group). (B) Survival analysis using TCGA dataset the results showed that high expression of ALX4 predicted longer survival time (n = 1080, *p* = 0.03, 3 group) **Table S1.** Primers used in this study. (DOCX 895 kb)

## Abbreviations

AlX4: Aristaless-like homeobox-4
BSP: Bisulfite genomic sequence
H&E: Hematoxylin-eosin staining
IHC: Immunohistochemistry
MSP: Methylation specific PCR
OS: overall survival
TCGA: The Cancer Genome Atlas
TMA: Tissue microarray analysis
