# Supplementary material for: ALX4, an epigenetically down regulated tumor suppressor, inhibits breast cancer progression by interfering Wnt/β-catenin pathway
Source: J Exp Clin Cancer Res. 2017 Nov 28;36:170. doi: 10.1186/s13046-017-0643-9 (PMC5706407; doi:10.1186/s13046-017-0643-9)
Supplement: Additional file 1: Figure S1. — The correlation between ALX4 expression and methylation was analyzed using TCGA data set (www.cbioportal.com). A: Cohort1:TCGA, Cell 2015, n = 552;Cohort2. B: TCGA, Provisional 2012, n = 656 Figure S2. Analysis the relationship between ALX4 and Wnt/β-catenin suppression and activation genes in breast cancer using TCGA Database. (A) ALX4 was positively correlated with WIF1 expression (R = 0.503, P = 0.00). (B) ALX4 was negatively correlated with LEF1 expression (R = −0.062, P = 0.03). (C) AXL4 was positively correlated with the expression of the key members of “β-catenin degradation complex” Figure S3. The illustration of full length human β-catenin promoter (−2760 bp to +27 bp) that was cloned into the PGL3-Basic luciferase reporter vector Figure S4. Relationship between ALX4 expression and survival of breast cancer patients retrieved from public datasets(https://genome-cancer.soe.ucsc.edu/proj/site/xena). (A) Survival analysis using TCGA dataset, the results showed that high expression of ALX4 predicted longer survival time (n = 1080, p = 0.00, 2 group). (B) Survival analysis using TCGA dataset the results showed that high expression of ALX4 predicted longer survival time (n = 1080, p = 0.03, 3 group) Table S1. Primers used in this study. (DOCX 895 kb) [file 13046_2017_643_MOESM1_ESM.docx]

**Additional file**

**Supplementary Figure legends**

Fig. S1 The correlation between ALX4 expression and methylation was analyzed using TCGA data set ([www.cbioportal.com](http://www.cbioportal.com)). A: Cohort1：TCGA, Cell 2015, n=552；Cohort2. B: TCGA, Provisional 2012, n=656.

Fig. S2 Analysis the relationship between ALX4 and Wnt/β-catenin suppression and activation genes in breast cancer using TCGA Database. (A) ALX4 was positively correlated with WIF1 expression (R = 0.503, P = 0.00). (B) ALX4 was negatively correlated with LEF1 expression (R = -0.062, P = 0.03). (C) AXL4 was positively correlated with the expression of the key members of “β-catenin degradation complex”.

Fig. S3 The illustration of full length human β-catenin promoter (-2760 bp to +27 bp) that was cloned into the PGL3-Basic luciferase reporter vector.

Fig. S4 Relationship between ALX4 expression and survival of breast cancer patients retrieved from public datasets（https://genome-cancer.soe.ucsc.edu/proj/site/xena）. (A) Survival analysis using TCGA dataset, the results showed that high expression of ALX4 predicted longer survival time (n=1080, p=0.00, 2 group). (B) Survival analysis using TCGA dataset the results showed that high expression of ALX4 predicted longer survival time (n=1080, p=0.03, 3 group).


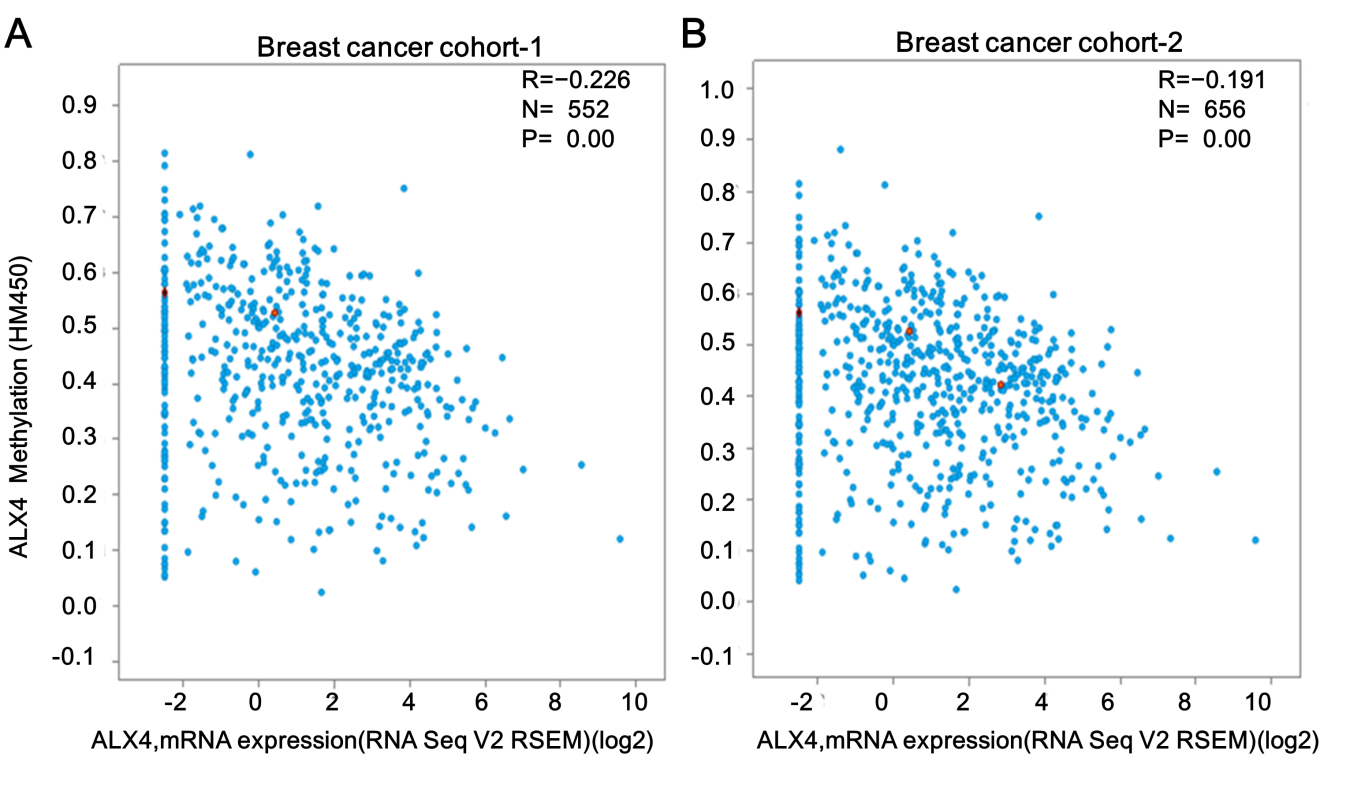


Fig. S1


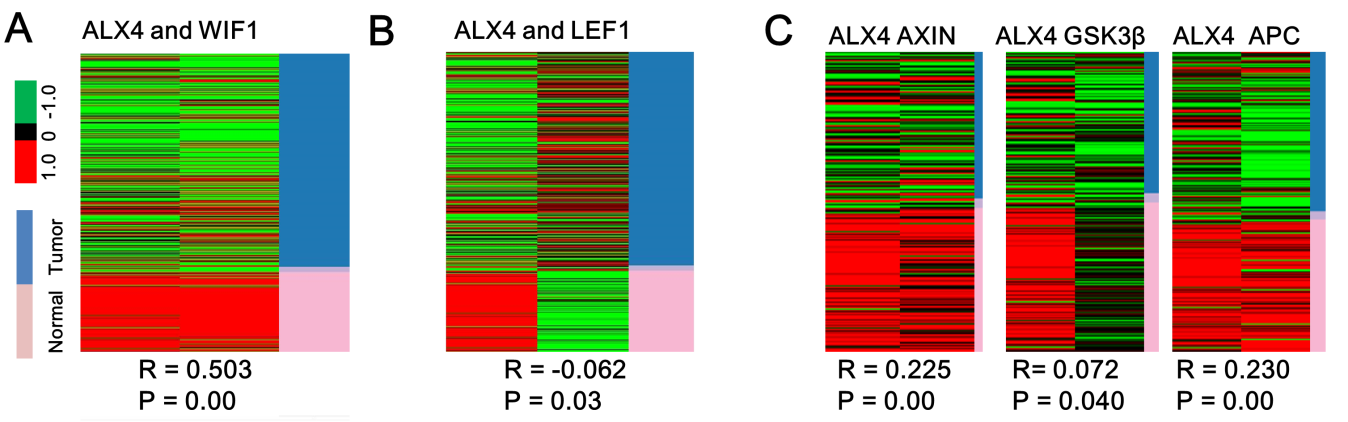


Fig. S2


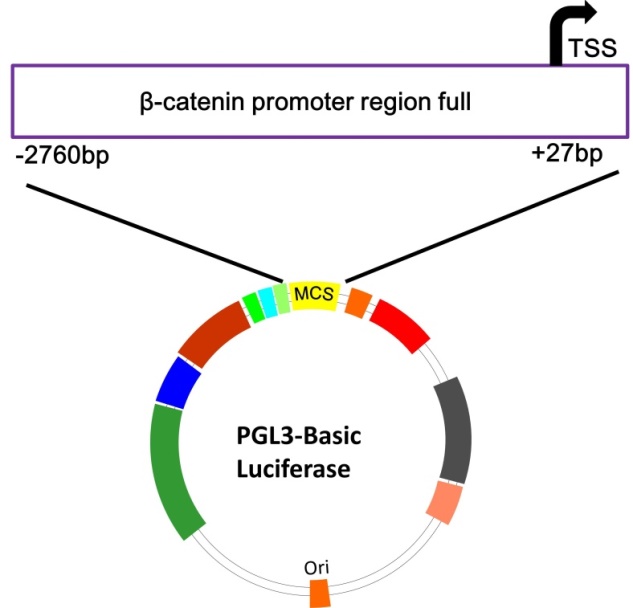


Fig. S3


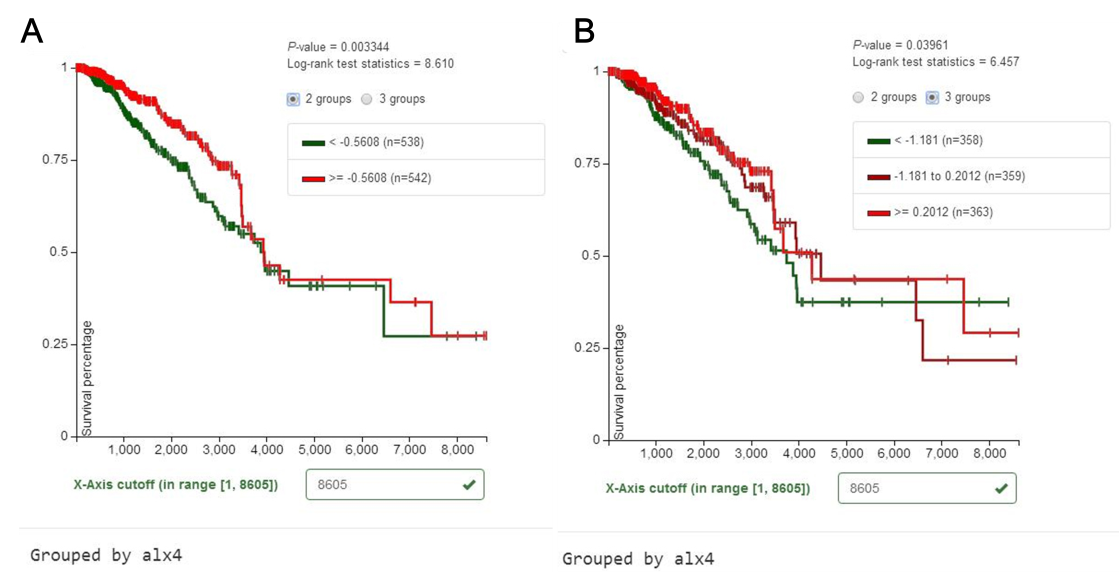


Fig. S4

**Supplementary Tables**

Table S1. Primers used in this study

| Primer name | Primer sequence 5'-3' | Primer purpose |
| --- | --- | --- |
| ALX4-qF | GCAGAACAGCTGCTACATCAAGAA | qPCR and RT-PCR |
| ALX4-qR | CAGTCGCTGGCGTAGATCTGTC | qPCR and RT-PCR |
| β-actin-qF | CCACGAAACTACCTTCAACTCC | qPCR and RT-PCR |
| β-actin-qR | GTGATCTCCTTCTGCATCCTGT | qPCR and RT-PCR |
| ALX4(MSP-M)-F | GTTAGGTATGAATGTTGAGATTTGC | MSP analysis |
| ALX4(MSP-M)-R | GAATCCCTATACTTTAACGACGAC | MSP analysis |
| ALX4(MSP-U)-F | TAGGTATGAATGTTGAGATTTGTGT | MSP analysis |
| ALX4(MSP-U)-R | CAAATCCCTATACTTTAACAACAAC | MSP analysis |
| ALX4(BSP)-F | AGTTAGGTATGAATGTTGAGATTTG | BSP analysis |
| ALX4(BSP)-R | AACTAAAACTTATTAAAAAAACCCC | BSP analysis |
| CTNNB1-F | GCGCCATTTTAAGCCTCTCG | qPCR and |
| CTNNB1-R | AAATACCCTCAGGGGAACAGG | qPCR |
| MMP7-qF | CATGATTGGCTTTGCGCGAG | qPCR |
| MMP7-qR | AGACTGCTACCATCCGTCCA | qPCR |
| MYC-qF | GCGAACACACAACGTCTTGG | qPCR |
| MYC-qR | TGAGCTTTTGCTCCTCTGCT | qPCR |
| CCND1-qF | GATGCCAACCTCCTCAACGA | qPCR |
| CCND1-qR | GGAAGCGGTCCAGGTAGTTC | qPCR |
| H-β_2_m F | GCTGTCTCCATGTTTGATGTATCTG | qPCR |
| H-β_2_m R | GCACGCTTAACTATCTTAACAAGCTTTG | qPCR |
| M-β_2_m-F | AGGCTTCTCTTTTTCTCCTCTGCTG | qPCR |
| M-β_2_m-R | TTTTCTCTCGACTTCGGTTGGATC | qPCR |
| CTNNB1-pF | GCTGCGATGCGGTACCGAACGAACCTTCTTTACAATACTGGCATAT | Promoter clone |
| CTNNB1-pR | GCGACTCGATCTCGAGACGCTGCTGCCACAGACCGAGAGGCTTAAAAT | Promoter clone |
